# Antibody Responses Against *Plasmodium vivax* TRAP Recombinant and Synthetic Antigens in Naturally Exposed Individuals From the Brazilian Amazon

**DOI:** 10.3389/fimmu.2019.02230

**Published:** 2019-09-20

**Authors:** Ada da Silva Matos, Rodrigo Nunes Rodrigues-da-Silva, Isabela Ferreira Soares, Barbara de Oliveira Baptista, Rodrigo Medeiros de Souza, Lana Bitencourt-Chaves, Paulo Renato Rivas Totino, Juan Camilo Sánchez-Arcila, Cláudio Tadeu Daniel-Ribeiro, César López-Camacho, Arturo Reyes-Sandoval, Lilian Rose Pratt-Riccio, Josué da Costa Lima-Junior

**Affiliations:** ^1^Laboratory of Immunoparasitology, Oswaldo Cruz Institute, Fiocruz, Rio de Janeiro, Brazil; ^2^Laboratory of Monoclonal Antibodies Technology, Institute of Immunobiology Technology, Fiocruz, Rio de Janeiro, Brazil; ^3^Laboratory of Malaria Research, Oswaldo Cruz Institute, Fiocruz, Rio de Janeiro, Brazil; ^4^Centre for Health Sciences and Sport, Federal University of Acre (UFAC), Cruzeiro Do Sul, Brazil; ^5^Viral Immunology Laboratory, Oswaldo Cruz Institute, IOC, Oswaldo Cruz Foundation, Fiocruz, Rio de Janeiro, Brazil; ^6^Nuffield Department of Medicine, The Jenner Institute, University of Oxford, Oxford, United Kingdom

**Keywords:** malaria, *Plasmodium vivax*, PvTRAP, humoral immune response, epitope mapping

## Abstract

Thrombospondin-related adhesive protein (TRAP) is essential for sporozoite motility and the invasion of mosquitoes' salivary gland and vertebrate's hepatocyte and is, thus, considered a promising pre-erythrocytic vaccine candidate. Despite the existence of a few reports on naturally acquired immune response against *Plasmodium vivax* TRAP (PvTRAP), it has never been explored so far in the Amazon region, so results are conflicting. Here, we characterized the (IgG and IgG subclass) antibody reactivity against recombinant PvTRAP in a cross-sectional study of 299 individuals exposed to malaria infection in three municipalities (Cruzeiro do Sul, Mâncio Lima and Guajará) from the Acre state of the Brazilian Amazon. In addition, the full PvTRAP sequence was screened for B-cell epitopes using *in silico* and *in vitro* approaches. Firstly, we confirmed that PvTRAP is naturally immunogenic in the cohort population since 49% of the individuals were IgG-responders to it. The observed immune responses were mainly driven by cytophilic IgG1 over all other sublcasses and the IgG levels that was corelated with age and time of residence in the studied area (*p* < 0.05). Interestingly, only the levels of specific anti-TRAP IgG3 seemed to be associated with protection, as IgG3 responders presented a significantly higher time elapse since the last malaria episode than those recorded for IgG3 non-responders. Regarding the B-cell epitope mapping, among the 148 responders to PvTRAP, four predicted epitopes were confirmed by recognition of antibodies (PvTRAP_R197−H227_; PvTRAP_E237−T258_; PvTRAP_P344−G374_; and PvTRAP_E439−K454_). Nevertheless, the frequency of responders against these peptides were low and did not show a clear correlation with the antibody response against the corresponding antigen. Moreover, none of the linear confirmed epitopes were located in the binding regions of PvTRAP in respect to the host cell ligand. Collectively, our data confirm the PvTRAP immunogenicity among Amazon inhabitants, while suggesting that the main important B-cell epitopes are not linear.

## Introduction

Malaria is caused by parasites belonging to the *Plasmodium* genus. Two species are considered the main etiological agents: *Plasmodium falciparum* and *P. vivax* (1). *P. falciparum* is more prevalent in Africa and is responsible for most cases of death around the world, and *P. vivax* is prevalent in the Americas (2). Despite several pre-clinical and a few clinical trials aiming for a global *P. vivax* vaccine development, there is still no licensed vaccine available.

The leading vaccine candidates against *P. vivax* are designed to tackle the pre-erythrocytic stage of lifecycle, and the sporozoite protein TRAP *(thrombospondin-related adhesive protein)* has been considered a promising candidate antigen. TRAP is a highly conserved transmembrane *Plasmodium* protein belonging to the TRAP/Micronemal protein 2 family (TRAP / MIC2). It is required for sporozoite motility and, in conjunction with the circumsporozoite (CS) protein, is essential for sporozoite invasion in the salivary gland of the mosquito and vertebrate hepatocytes (3, 4). Structurally, TRAP contains an N-terminal hydrophobic sequence (domain I), an integrin A-type magnesium binding Domain (domain II), a thrombospondin type I replications (domain III), an acid proline/asparagine domain region (IV), a hydrophobic transmembrane domain (V domain), and a cytoplasmic tail region (5). Sporozoite locomotion is mediated by the subpellicular actomyosin system that binds to the cytoplasmic tail of TRAP (6). Despite the functional importance of TRAP in parasite survival, analysis of *P. falciparum* TRAP (PfTRAP) and *P. vivax* (PvTRAP) loci of clinical isolates revealed a small sequence heterogeneity (7).

The potential of TRAP as vaccine antigen is currently supported by several reports of immunogenicity and protection in mice, non-human primates and humans. For instance, in a murine model, co-immunization of CSP with TRAP provided complete protection against a parasite challenge, whereas vaccination using only CSP provided only partial protection (8). In monkeys, immunization with synthetic peptides representing domain II of PvTRAP was able to protect 2/3 of the challenged animals with *P. vivax* sporozoites (9). In humans, Phase IIa clinical trials with PfTRAP showed complete sterile protection in more than 20% of vaccinated individuals and delayed patency in 58% (10). In fact, TRAP-specific CD8+ T lymphocytes seem to be key mediators in the protection against sporozoite challenge in mouse vaccination assays (11) and in clinical trials with PfTRAP-vaccinated human individuals (10). However, antibodies are also important players in protection due to its activity on sporozoite motility/invasion blocking (12, 13) and, in this context, recent sero-epidemiological studies showed that anti-PfTRAP antibodies were negatively correlated with parasite density among infected individuals in malaria endemic areas (14, 15).

However, there is still little knowledge about TRAP natural antigenicity in *P. vivax*-exposed populations. Epidemiological, genetic, and environmental factors can influence the development of immunity against malaria parasites, and the understanding of humoral immune responses is relevant because the knowledge of the specific patterns of antibodies response across different endemic areas worldwide can improve the vaccine development process (16). Moreover, since antibodies recognize antigens through their binding to B-cells epitopes, the prediction of epitopes recognized by the immune humoral response can allow the development of epitope-based vaccines (17–19). Our present study provides, for the first time, an evaluation of the antibody response to PvTRAP in individuals of the Brazilian Amazon, by characterizing the specific profile of PvTRAP IgG subclasses antibodies and identifying linear B-cell epitopes that participate in the natural immune acquired response. Results are discussed along with data on individual exposure and protection.

## Materials and Methods

### Study Area and Volunteers

**Figure 1:**
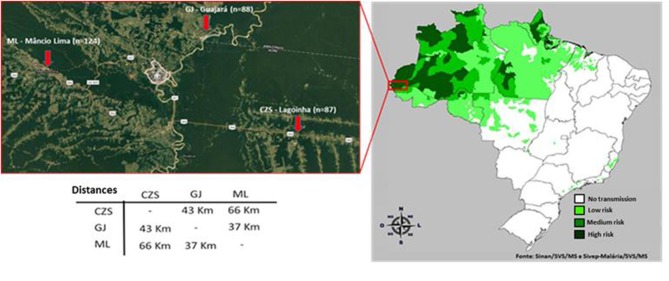
Areas of samples collection. The three areas of sample collection in the north of Brazil (state of Acre) are approximately fifty kilometers apart from each another. CZS, Cruzeiro do Sul; GJ, Guajara; and ML, Mancio Lima.

A cross-sectional cohort study was conducted involving 352 individuals from three different endemic areas of Acre (Figure 1): Cruzeiro do Sul (*n* = 124), Guajará (*n* = 87), and Mâncio Lima (*n* = 88). Fifty-three samples from individuals living in non-endemic areas from Rio de Janeiro were considered the control group. Samples and survey data were collected from June 2016 to August 2016. Written informed consent was obtained from all donors and the study was reviewed and approved by the Oswaldo Cruz Foundation Ethical Committee and the National Ethical Committee of Brazil (CEP-FIOCRUZ CAAE 46084015.1.0000.5248).

### Epidemiological Survey

In order to evaluate the possible influence of epidemiological factors on humoral immunity against PvTRAP, all donors were interviewed. The survey included questions related to personal exposure to malaria, such as years of residence in the endemic area, recorded individual and family previous malaria episodes, use of malaria preventive measures, presence/absence of symptoms, and personal knowledge on malaria transmission. The volunteers answered the questions and all epidemiological data were stored in Epi-Info (Centers for Disease Control and Prevention, Atlanta, GA, USA) for subsequent analysis.

### Malaria Diagnosis and Blood Sampling

Peripheral blood samples were collected by venipuncture in heparin tubes. After centrifugation (350 × g, 10 min), the plasma was collected and stored at −20°C and transported to our laboratory. Thin and thick blood smears of all donors were examined for malaria parasites. Parasitological evaluation was done by examination of 200 fields at 1,000× magnification under oil-immersion and a research expert in malaria diagnosis examined all slides. All donors found positive for *P. vivax* and/or *P. falciparum* at blood smears were subsequently treated using the chemotherapeutic regimen recommended by the Brazilian Ministry of Health.

### Recombinant PvTRAP

For the expression and purification of PvTRAP protein, the codon-optimized gene of *P. vivax* TRAP (UniProt A5K806, residues Asp25 to Lys493) was cloned into the pHLsec vector (20), which is flanked by the chicken β-actin/rabbit β-globin hybrid promoter with a signal secretion sequence and a Lys-His6 tag. Endogenous PvTRAP secretion signal (a.a. 1-24) was replaced by a signal sequence contained in the PHLsec mammalian expression plasmid (MGILPSPGMPALLSLVSLLSVLLMGCVA). In order to improve secretion of the PvTRAP protein, the C-terminal region of PvTRAP (a.a. 494-556) was deleted. The pHLsec PvTRAP plasmid (500 μg) was transfected in HEK-293T cells using polyethyleneimine (PEI) in roller bottles (surface area of 2,125 cm^2^) under standard cell culture conditions. Five days after transfection cells were discarded and media was filtered through 0.22 μM disposable filters. The secreted protein was purified from the supernatant by Nickuel Sepharose affinity chromatography (HisTRAP™, GE Healthcare), using the Äkta Start chromatography system and eluted with Imidazole 500 mM. Finally, eluted protein was dylazed using Slide-A-Lyzer™ cassette (Fisher Scientific) against 1X PBS.

### ELISA

Antibody binding to PvTRAP was evaluated on plasma samples by enzyme-linked immunosorbent assay (ELISA). Briefly, MaxiSorp 96-well-plates (Nunc, Rochester, NY, USA) were coated with PBS containing 1.5 μg/ml of recombinant PvTRAP. After overnight incubation at 4°C, the plates were washed with PBS-Tween and blocked for 1 h at 37°C. Plasma samples diluted 1:100 in PBS-Tween containing 5% non-fat dry milk (PBS-Tween-M) were added in duplicate wells for each individual. After 1 h at 37°C and three washings procedures, specific antibodies were detected by peroxidase-conjugated anti-human IgG (Sigma, St. Louis) and followed by the addition of o-phenylenediamine and hydrogen peroxide. Optical density was accessed at 490 nm using a SpectraMax 250 ELISA reader (Molecular Devices, Sunnyvale, CA, USA). Results for total IgG were expressed as reactivity indexes (RIs), which represent the mean optical density of each tested sample divided by the mean optical density of 10 non-exposed control individuals' samples plus 3 standard deviations. Subjects were scored as responders to PvTRAP if the RI of IgG against the recombinant protein was higher than 1. Additionally, IgG subclasses were evaluated on responders by the same method, using peroxidase-conjugated goat anti-human IgG1, IgG2, IgG3, and IgG4 (clone HP-6001 for IgG1, HP-6002 for IgG2, HP-6050 for IgG3, HP-6023 for IgG -Sigma, St. Louis).

### B-Cell Epitope Prediction

To predict linear B-cell epitopes on entire sequences of PvTRAP (U64901.1; Salvador 1), we used a combination of three prediction algorithms: BCpreds (http://ailab.ist.psu.edu/bcpred/predict.html), BepiPred1.0 (http://www.cbs.dtu.dk/services/BepiPred-1.0/), and ABCpred (http://crdd.osdd.net/raghava/abcpred). BepiPred 1.0 (21) is based on hidden Markov model profiles of known antigens, and also incorporates hydrophobicity and secondary structure prediction. We used the recommended cutoff of 0.90 to determine potential B-cell linear epitopes. Furthermore, ABCpred and BCpreds are both based learning methods of the machine. In this study, we considered threshold values of 0.3, 0.51, and 75% to BepiPred, ABCpred, and BCpreds, respectively. All sequences with more than 10 amino acids and predicted for at least two of prediction algorithms were considered as a linear B-cell epitope and evaluated by antigenicity using the Vaxijen algorithm (http://www.ddg-pharmfac.net/vaxijen/VaxiJen/VaxiJen.html). VaxiJen is the first server for alignment-independent prediction of protective antigens. It was developed to allow antigen classification solely based on the physicochemical properties of proteins without recourse to sequence alignment. Bacterial, viral and tumor protein datasets were used to derive models for the prediction of whole protein antigenicity, showing prediction accuracy from 70 to 89% (22). To evaluate the antigenicity of predicted epitopes we used the threshold of 0.6. All predicted epitopes with a Vaxijen score higher than threshold value (0.6) were selected to experimental validation.

### Peptide Synthesis

After a consensual analysis of the *in silico* prediction tools, four peptide sequences appeared as relevant epitopes within PvTRAP. Therefore, the sequences were synthesized by fluorenylmethoxycarbonyl (F-moc) solid-phase chemistry (23) (GenOne Biotechnologies, Brazil). Analytical chromatography of the peptides demonstrated a purity of >95% and the mass spectrometric analysis also indicated the estimated masses for each of them: 3395.78 g/mol (peptide PvTRAP_R197−H227_), 2332.61 g/mol (peptide PvTRAP_E237−T258_), 3487.59 g/mol (peptide PvTRAP_P344−G374_), and 2333.55 g/mol (peptide PvTRAP_E439−I460_).

### Confirmation of B-Cell Epitopes (Peptide ELISA)

To confirm the overlapping epitopes detected with the prediction programs, enzyme-linked immunosorbent assay (ELISA) was performed. MaxiSorp 96-well-plates (Nunc, Rochester, NY, USA) were coated with PBS containing 0.5 μg/ml of each peptide, followed by overnight incubation at 37°C. The plates were washed and blocked with BSA 4% for 1 h and 30 min at 37°C in the humid incubator. After this, the individual plasma samples were diluted 1:100 in PBS-Tween containing BSA 2% in duplicate wells. After 2 h at 37°C in the humid incubator, and three washings with PBS-Tween, bound antibodies were detected with peroxidase-conjugated goat anti-human IgG (Sigma, St. Louis) and followed by the addition of o-phenylenediamine and hydrogen peroxide. Optical density was identified at 490 nm using a SpectraMax 250 ELISA reader (Molecular Devices, Sunnyvale, CA, USA). As made for recombinant protein, the results for each peptide were expressed as reactivity indexes (RIs), which were calculated by the mean optical density of an individual's tested sample divided by the mean optical density of 10 non-exposed control individuals' samples plus 3 standard deviations.

### Statistical Analysis

All statistical analyses were carried out using Prism 5.0 for Windows (GraphPad Software, Inc.). The one-sample Kolmogorov–Smirnoff test was used to determine whether a variable was normally distributed. The Mann–Whitney test was used to compare RIs of IgG against recombinant PvTRAP between studied groups. Differences in proportions of the RI of IgG subclasses and epidemiological parameters were evaluated by Fisher's exact test and associations between antibody responses and epidemiological data were determined by Spearman rank test. A two-sided *p* < 0.05 was considered significant.

We used a principal component analysis (PCA) to detect the variation pattern of Anti-PvTRAP total IgG, age, time of residence in endemic area, time since last infection response, and number of previous malaria infections. Our objective was to reduce dimensionality of multivariate data to detect the variables that explained the variance structure of the data. Each axis of PCA, hereafter named Principal Component (PC) represents the amount of variation associated to this axis. Each variable studied in the PCA has a loading value indicating its contribution to each PC. In the graph, individuals are represented as points located between principal component 1 (PC1) and principal component 2 (PC2). Variables are represented as arrows and its longitude represent its contribution to the variation of data. Additionally, closer angles between variables (arrows), represents more correlated variables, and angles closer to 90° represent independence between variables (orthogonality).

To evaluate total IgG responses to PvTRAP peptides and compare with PvTRAP recombinant protein, we constructed a two-dimensional heatmap with hierarchical clustering grouping individuals with similar responses and the responses for peptides and recombinant PvTRAP. We calculated Z-scores from transformed total IgG values for PvTRAP peptides and recombinant protein: Z-score = [(PvTRAP peptides/recombinant protein total IgG values – population PvTRAP peptides/recombinant protein total IgG mean value)/population PvTRAP peptides/recombinant protein total IgG standard deviation]. Clustering was performed from Z-scores using Euclidean distance metrics and Ward as the linkage algorithm. PCA was constructed using vegan package (24) and a two-dimensional heatmap was build using gplots package (25) in R statistical environment (26).

## Results

### Epidemiological Profile of Studied Individuals

The studied population is composed of 299 individuals living in three close municipalities of the Brazilian Amazon. The majority was composed by adults and all individuals were naturally exposed to malaria infection (Table 1). The age range was 12–88 years with an average of 35 years, and the cohort presented a similar prevalence in gender (male = 48.3%; female = 51.7%). The time of residence in malaria endemic area (TREA) ranged from 3 to 88 years, which indicated different degrees of exposure among the studied individuals. With regards to prior history of malaria infections, 11% of all studied individuals did not remember having had previous malaria episodes (PME) and 2% did not report any malaria episode during their entire life. Sixty-two percent informed us of previous episodes of *P. falciparum* and *P. vivax* malaria during their entire life, which are the two prevalent species in Brazil. The number of past infections reported by individuals also varied greatly, ranging from 0 to 48 (mean = 8.62 ± 10.24) and the time elapsed since the last infection (TLI) varied from 0 to 240 months (mean = 33.5 ± 57.1). Finally, at the time of blood collection, 118 (39.5%) individuals were naturally infected with *Plasmodium* sp. The frequency of *P. vivax* infections (24.4%) was significantly higher than that of *P. falciparum* infections (13.7%; *p* = 0.0016). Collectively, the parameters evaluated indicated that the studied population are naturally exposed to malaria, but have different degrees of exposure and/or immunity.

**Table 1:** Summary of the epidemiological characteristics of studied individuals enrolled in the survey.

**Epidemiological features**	**Total**
	**(*****N*** **=** **299)**
**Gender**	***N*** **(%)**
Male	48.3%
Female	51.7%
**Malaria exposure**	**Mean (±SD)**
Age	35 (±15.97)
Time of residence in the endemic area	34 (±16.10)
Time of residence in the present address	24 (±19.35)
Months since the last malaria episode	33 (±57.07)
Number of malaria episodes on the last year	0.82 (±1.13)
Number of previous malaria episodes	10 (±12.41)
**Previous species contracted**	***N*** **(%)**
*P. vivax*	56 (19%)
*P. falciparum*	16 (5%)
*P. vivax and P. falciparum*	186 (62%)
Never infected	7 (2%)
Not reported	34 (11%)
**Diagnosis**	***N*** **(%)**
*P. vivax*	73 (24.4%)
*P. falciparum*	41 (13.7%)
Mixed	4 (1.3%)
Negative	181 (60.5%)
**Species of the last infection**	***N*** **(%)**
*P. vivax*	173 (48%)
*P. falciparum*	62 (17%)

*Values of age, time of residence in endemic areas (TREA) and time of residence in the present address (TRPA), months since the last malaria episode (MSLM), number of malaria episodes on the last year, and number of previous malaria episodes (NPME) are represented by mean (with standard variation values). The frequency of individuals regarding to malaria diagnosis, previous infections and species of the last infection was compared by Fisher's test, and other epidemiological parameters were compared by Mann–Whitney test. We can observe that the prevalence of P. vivax cases is significantly higher than that of P. falciparum cases (p < 0.0001)*.

### Frequency and Magnitude of IgG Immune Response Against Recombinant PvTRAP

**Figure 2:**
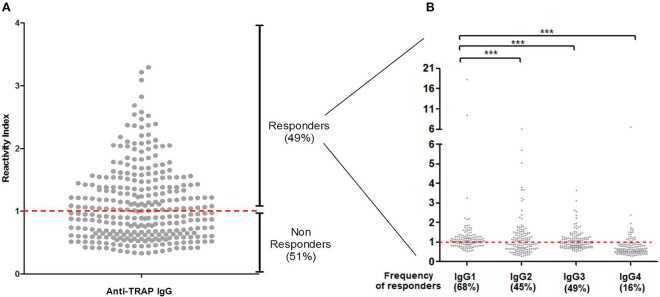
**(A)** Frequency of responders and reactivity indices of specific IgG antibodies. The red line represents the cutoff reactivity index (RI) value that was considered to classify individuals as positive (>1) and negative (<1) for PvTRAP. **(B)** Profile of IgG subclasses in responder individuals for PvTRAP. ***p < 0.0001.

To verify if PvTRAP is a target of naturally acquired humoral response against *P. vivax* in Brazilian amazon individuals, we assessed the IgG reactivity against the recombinant antigen. Firstly, we confirmed that PvTRAP is immunogenic in naturally exposed individuals from the Brazilian Amazon, as 148 (49%) individuals presented specific antibodies against the recombinant PvTRAP (Figure 2A) with no significant difference among *P. vivax* or *P. falciparum* infected individuals and exposed but non-infected individuals (Figure S1). Among responders, the reactivity indexes (RI) values ranged from 1.01 to 4.29 (mean = 1.55 ± 0.48). We also assessed the overall distribution of the IgG antibody subclass responses to PvTRAP protein using different comparative analyses. Secondly, we determined subclass-specific prevalence in total IgG positive responders, in which IgG1 was the most prevalent subclass present in 68% of responders (Figure 2B), followed by IgG3 (49%) and IgG2 (45%). The subclass with the lower frequency was IgG4 (16%). The frequency of IgG1 responders was significantly higher when compared to IgG2 (*p* = 0.0002), IgG3 (*p* = 0.0021), and IgG4 (*p* < 0.0001). Thirdly, in relation to magnitude of antigen-specific IgG subclass responses, a wide distribution of RIs was observed, which ranged from 0.28 observed in IgG2 to 18.33 in IgG1. However, the overall mean of specific subclass RIs indicate that IgG1 cytophilic antibodies against PvTRAP (mean = 1.53 ± 1.91) was also significantly higher (*p* < 0.0001) than all other subclasses (IgG2 = 1.17 ± 0.93, IgG3 = 1.08 ± 0.47), and IgG4 (mean = 0.76 ± 0.60).

### Influence of Malaria Exposure on Naturally Acquired IgG Against PvTRAP

**Figure 3:**
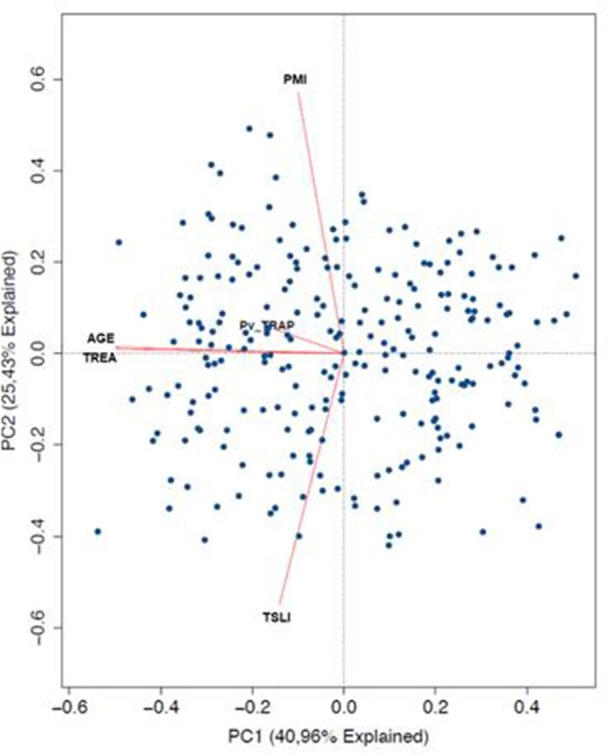
Principal Component analysis of IgG Anti-PvTRAP and epidemiological variables. Representation of principal components 1 (PC1 = 40.96% and 2 PC2 = 25.43%). Each point in the graphs represents a single individual along multidimensional space. The length of the arrows represents the strengh in variability explaining for each principal component. (TREA, time of residence in the endemic area; PMI, past malaria infections; TSLI, time since the last malaria infection/episode).

**Figure 4:**
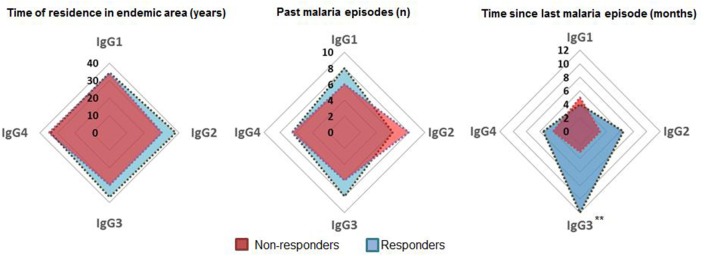
Comparison of exposure parameters between responders and non-responders for IgG subclasses. Medians of time of residence in endemic area (TREA), past malaria episodes (PME), and past months since last infection (TLME) for positive and negative individuals tested for IgG and subclasses were compared. Positive individuals tested for IgG3 presented a higher median of TLME. **p < 0.01.

In order to identify the contribuition of epidemiological data on the variation observed in humoral response to PvTRAP, data was matched with the magnitude and frequency of responses to PvTRAP. Firstly, we noted that the distribution of individuals into ordination space was homogeneous, without evident groups into it. Age and TREA were highly correlated, and previous malaria infections as well as time since last malaria infection were negatively correlated between them. However, only age and TREA was related to RIs against PvTRAP IgG to data variation. The nature of variables relationship was confirmed by calculating the correlation between the anti-PvTRAP IgG reactivity index and age (*r* = 0.190; *p* = 0.001) and TREA (*r* = 0.194; *p* = 0.001). Antibody levels and the number of previous infections were orthogonal variables and we also did not observe correlation (*r* = 0.013; *p* = 0.832). A similar result was observed in the PCA for anti-PvTRAP IgG reactivity index and time elapsed since the last malaria episode (*r* = −0.051; *p* = 0.398) (Figure 3). Regarding the IgG subclass and malaria exposure and/or indicatives of protection, there was no significant difference between subclass responders and non-responders in relation to TREA or PME. However, interestingly, the time elapsed since the last malaria episode was significantly higher in TRAP-specific IG3 responders than non-responders (Figure 4). This finding was corroborated by the observed correlation between IgG3 RIs against PvTRAP and the time elapsed since the last malaria episode (*r* = 0.184; *p* = 0.0032).

### *In silico* Prediction of B-Cell Linear Epitopes of PvTRAP

**Figure 5:**
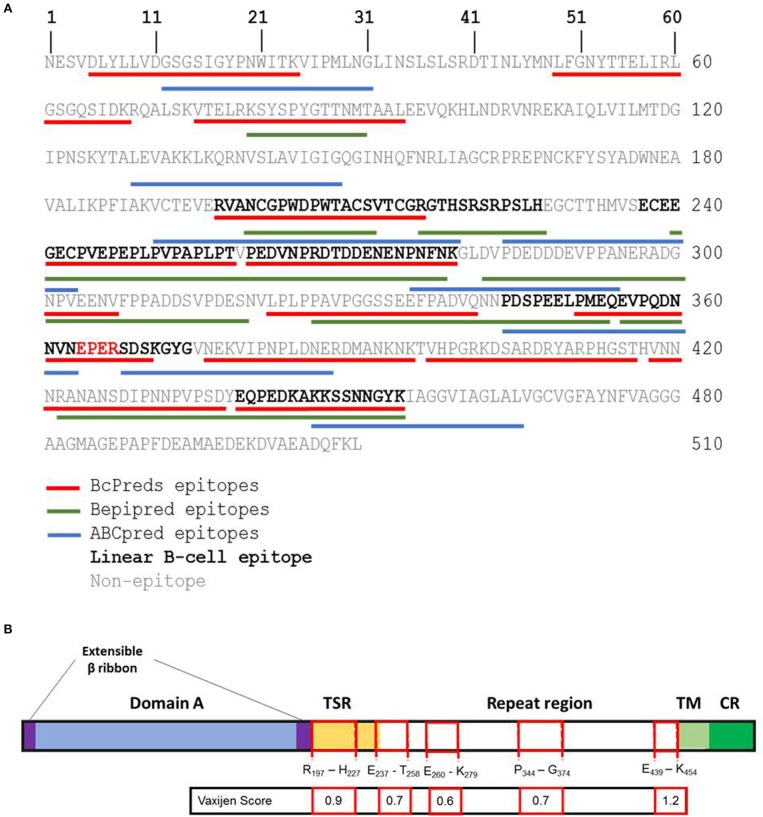
*In silico* identification of linear epitopes on PvTRAP. **(A)** *In silico* prediction of linear epitopes on PvTRAP sequence. Letters on figure represent one letter amino acid code. Bcpreds, Bepipred, and ABCpred predicted epitopes were represented by red, green, and blue lines, respectively. Black bold letters indicate predicted epitopes. Sequences with more than 9-mers and predicted by at least two algorithms were considered B-cell linear epitopes. **(B)** Schematic diagram of PvTRAP and predicted linear B-cell epitopes. The black line box represents the PvTRAP. The blue box represents the domain A, the purple boxes are Extensible β ribbon, the yellow box represent the thrombospondin type 1 repeat (TSR) domain, the white box represents the repeat region, the light green box is the transmembrane region (TR) and dark green represents the cytosolic region (CR) of PvTRAP. Predicted linear B-cell epitopes were illustrated by red line boxes and the first and the last amino acids of each epitope were indicated. The Vaxijen Score indicates the value of predicted antigenicity of each epitope.

Firstly, five sequences were predicted as potential linear B-cell epitopes on PvTRAP (PvTRAP_R197−H227_, PvTRAP_E237−T258_, PvTRAP_P260−K279_, PvTRAP_P344−G374_, and PvTRAP_E439−K454_) (Figure 5A). Among these sequences, four (PvTRAP_R197−H227_, PvTRAP_E237−T258_, PvTRAP_P344−G374_, and PvTRAP_E439−K454_) presented Vaxijen score higher than threshold value (0.6) and were considered antigenic epitopes selected to experimental validation. Regarding the localization of predicted antigenic linear B-cell epitopes, epitopes PvTRAP_E237−T258_; PvTRAP_P344−G374_, and PvTRAP_E439−K454_ were localized in repeat region of TRAP, while epitope PvTRAP_R197−H227_ was inserted in thrombospondin type I repeat (TSR) domain (Figure 5B).

### Experimental Validation of Predicted Linear B-Cell Epitopes

To validate predicted antigenic epitopes of PvTRAP, we tested by ELISA the plasma from responders to the recombinant protein, against selected epitopes (PvTRAP_R197−H227_, PvTRAP_E237−T258_, PvTRAP_P344−G374_, and PvTRAP_E439−K454_).

**Figure 6:**
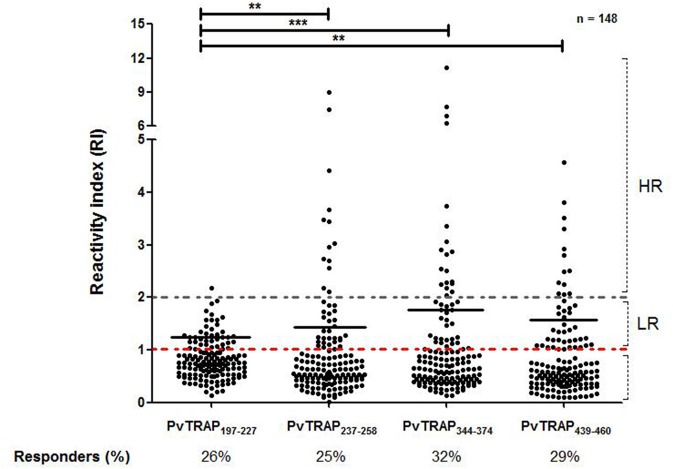
Frequency of responders for tested peptides and mean of RI among responders (low responders and high responders). Each point represents an individual RI against PvTRAP and the red traced line represents the cutoff that separates responders and non-responders. The gray dotted line separates the individuals tested in low responders (LR) (RI>1 and RI<2) and high responders (HR) (RI>2). Peptide PvTRAP_R197−H227_presented a lower median of RIthan peptide PvTRAP_E237−T258_ (*p* = 0.0049), peptide PvTRAP_P344−G374_ (*p* < 0.0001), or peptide PvTRAP_E439−K454_ (*p* = 0.0073).

Among the 148 responders to PvTRAP recombinant protein, as showed in Figure 6, all predicted epitopes (PvTRAP_R197−H227_, PvTRAP_E237−T258_, PvTRAP_P344−G374_, and PvTRAP_E439−K454_) presented low frequencies of responders (26, 25, 32, and 29%, respectively). Beside, peptide R_197_-H_227_ presented the lowest magnitude of response (median = 1.25 interquartile range = 1.13–1.47) when compared to PvTRAP_E237−T258_, PvTRAP_P344−G374_, or PvTRAP_E439−K454_ (median = 1.60 interquartile range = 1.22–2.65, *p* = 0.003; median = 1.87 interquartile range = 1.12–2.61, *p* < 0.0001; median = 1.44 interquartile range = 1.16–2.08, *p* = 0.035; respectively).

In order to investigate the protective role of antibodies against linear epitopes of PvTRAP, we compared epidemiological data from individuals who recognized at least one of the linear epitopes with data from those who did not recognize linear epitopes. Interestingly, individuals who were responders to linear epitopes presented a lower mean of the time elapsed since the last malaria episode (mean = 23.97 ± 49.78) and a higher number of previous malaria episodes (mean = 13.35 ± 14.10) when compared to non-responders (mean = 40.20 ± 63.91, *p* = 0.0175; mean = 8.35 ± 9.60, *p* = 0.005, respectively).

**Figure 7:**
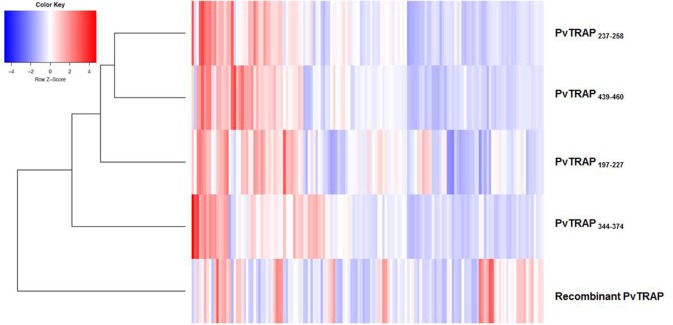
Clustered heatmap. Each cell represents the IgG value for the studied PvTRAP peptides and recombinant protein. The vertical cluster shows the grouping hierarchy of PvTRAP peptides and recombinant protein. Along horizontal axis, individuals with similar responses are grouped. The red color in the cells indicates high IgG values and the blue color indicates low ones. The white color represents no changes in the IgG values for PvTRAP peptides and recombinant protein.

In order to understand the profile of IgG directed to peptides and its relationship to IgG response detected agains recombinant PvTRAP, we used a clustered bidimensional heatmap (Figure 7). In the vertical cluster localized in the left side of the heatmap, we noted that those peptides grouped in the first level of the cluster. PvTRAP_E237−T258_ and PvTRAP_E439−k460_ presented more similarity followed by PvTRAP_R197−H227_ and PvTRAP_P344−G374_. The IgG responses were heterogeneous. IgG values for peptides were more similar between them compared to recombinant PvTRAP. Nevertheless, responses for recombinant PvTRAP were heterogeneous and not matched with high RI values for peptides. We also observed a mixed profile of responses including individuals that responded to recombinant PvTRAP but not to peptides, other non-responders to peptides and recombinant PvTRAP, and individuals that responded to peptides and recombinant PvTRAP.

## Discussion

Despite numerous advances in the understanding of the biology of *Plasmodium vivax*, there is still no vaccine against this parasite. Recently, PvTRAP has emerged as a leading vaccine candidate (27). Sero-epidemiological studies have played a significant role in the identification of leading vaccine candidates (28), which underlies the importance of assessing the immune response against PvTRAP and its role in immunity in exposed individuals from endemic regions, such as within the Brazilian Amazon. Here, we have assessed the naturally acquired humoral immune response against a recombinant PvTRAP and have investigated the associations between TRAP-specific immune responses with the epidemiological profile of inhabitants of three municipalities in the southwestern Brazilian Amazon. We additionally searched for linear B-cell epitopes in the entire PvTRAP sequence and associated the immune response against epitopes and the recombinant protein.

The profile of studied individuals indicates that our population is composed of rainforest region natives and migrants from non-endemic areas of Brazil who had lived in the area for more than 10 years (92%). The majority of individuals reported a prior exposure to *P. vivax* and *P. falciparum* malaria parasites. The highly variable range of numbers of previous infections, the time of residence in endemic area, and the time elapsed since the last infection, suggest that there are differences in exposure and immunity. It is well-known that the acquisition of clinical immunity is mediated by antibodies and depends on continued exposure to the parasite (29, 30). Selection of individuals in this cohort from the Amazon was ideal to assess presence of antibodies against the PvTRAP and distinguish whether their relationship to malaria exposure and/or indicatives of protection.

Firstly, we confirmed that PvTRAP is naturally immunogenic in individuals from the Amazon, since almost half of volunteers studied presented TRAP-specific IgG antibodies with a wide range in magnitude of IgG response. Our results were similar to TRAP reactivity in South East Asia, where Kosuwin et al. found an overall 51% reactivity against PvTRAP Domain II in Thailand in exposed individuals (31), and in middle-east endemic areas, where Nazeri et al. found an immune response with related frequencies in Iran (42%), Afghanistan (38%), and Pakistan (44%) (16). The frequency of responders to PvTRAP in Brazilian endemic areas herein reported was also comparable to other *P. vivax* vaccine candidates, such as AMA-1 (32), MSP-9 (33), MSP-1 (34), and DBP (35) in Amazon regions, as well as to PfTRAP in unstable transmission endemic areas of Africa (36) and Iran (37). Despite the naturally acquired immune response against PvTRAP being confirmed in studied areas, we did not find differences in reactivity index of IgG antibodies among exposed individuals with *P. falciparum, P. vivax* and negatives. As expected, such differences are most noticeable in serology against erythrocyte phase antigens, since individuals residing in the transmission areas may be constantly receiving infectious stimuli by sporozoites that could not develop the disease but still stimulate the immune system and the production of specific antibodies. It is important to mention that recently a frequency of uninfected responders against PfCSP and PvCSP derived antigens was also reported in Brazilian endemic areas (38).

Studies on the humoral immune responses to *P. falciparum* antigens have constantly shown that immunity to blood-stage antigens is dependent on a specific pattern of immunoglobulin subclass response. Pattern of IgG subclasses responses of IgG1 and/or IgG3 antibodies to several blood-stage antigens are effective mediators of antibody-dependent cellular inhibition (ADCI) of malaria parasites growth *in vitro*, associated with the acquisition of clinical immunity to malaria (39–42) and with fixation and complement activation (43). In fact, the subclass-specific antibodies to *P. vivax* in the development of protective immunity is still unclear and the effect of each IgG subclass is still controversial. In our cohort, the IgG subclass profile among responders revealed an IgG1 biased humoral response with a considerable frequency of IgG3 and IgG2 antibodies. The prevalence of cytophilic antibodies against TRAP was also observed in individuals from the Middle East. However, the high frequency of IgG2 found in our work was not observed in individuals from those studies (16). In fact, we can hypothesize that host genetics factors, such HLA polymorphisms, can affect the development of specific immune response against PvTRAP as it does against other *P. vivax* antigens (44). In addition, epidemiological and environmental factors certainly affect the immune repertoire activation, potential of recognition and magnitude of TRAP-specific antibodies in such different regions. Therefore, the parameters of exposure and/or protection available in our epidemiological survey were matched with the magnitude of IgG specific antibodies in order to allow the study of potential associations. Regarding exposure, age, and time of residence in endemic areas were correlated with magnitude of anti-PvTRAP IgG antibodies. However, since in our study the majority of individuals were born in endemic areas, age related changes in antibody responses could reflect, in fact, only the time of exposure in an endemic region. This phenomenon has been frequently reported for various antigens (45–48) and most likely could reflect exposure to the malaria parasite and possibly maturation of the immune system over time, which can culminate in protection against subsequent infections. Unfortunately, the cross-sectional design of our study limited the investigation to retrospective malaria histories reported by the volunteers, and the best approximation of an individual's protection was the time elapsed since their last recalled malaria episode. Using this approach, we observed that IgG3 responders presented a significantly longer time since the last malaria episode than IgG3 non-responders. These results could suggest a possible role of PvTRAP cythophilic antibodies in protective immunity against *P. vivax* infection. However, more prospective studies on humoral immune responses with a follow up of participants and/or biologic studies addressing the ability of these antibodies to inhibit sporozoite motility or invasion will provide more direct evidence in protective efficacy of specific PvTRAP antibodies.

Whilst there is strong evidence showing that specific antibodies are associated with cumulative exposure and protection against vivax malaria (28, 30, 49–51), the relative contribution of different regions to the molecule inducing protective antibodies is another important point to be addressed. Therefore, we also map the linear epitopes in PvTRAP using *in silico* tools and confirmed the antigenicity by ELISA in PvTRAP responders. The four linear epitopes in PvTRAP sequence were identified in different regions of protein (PvTRAP_R197−H227_ in the TSR domain; PvTRAP_E237−T258_ in a region between the repeat region and TSR domain; PvTRAP_P344−G374_ fully in the repeat region; and PvTRAP_E439−I460_ in the repeat region near to the transmembrane domain). It is important to mention that two extracellular portions of PvTRAP (Domain II and repetitive region) are crucial for initial host cell adherence and stabilization of adhesion/deadhesion during gliding mobility of sporozoites (52) and are associated with protective immunity against *P. falciparum* (50). In our work, no linear epitope was confirmed in Domain II and the epitopes located at repetitive region presented low frequency of reactivity among responders to full-length recombinant PvTRAP. The frequencies and magnitude of antibodies against PvTRAP-derived peptides were also lower than other linear epitopes identified in sporozoite surface of *P. vivax*, such CelTOS (53) and CSP (54, 55), and merozoites (MSP9 and AMA-1) (19, 56). Indeed, despite the observation of a discrete immunodominance of PvTRAP_P344−G374_ fragment over the other peptide epitopes, the heatmap analysis and the correlation of reactivity index against recombinant protein did not show a possible relationship with epidemiological parameters related to exposure or protection. The Domain II folded region probably presents conformational epitopes, which seem to be more related with blocking activity and protective immunity, making it a more suitable candidate for vaccine development. Therefore, the functional roles of these specific antibodies need to be further investigated. Lastly, although we have found a high response against PvTRAP, we do not believe that there is a cross response between PfTRAP and PvTRAP as the amino acid sequence of these proteins has low homology and prediction of epitopes for both proteins revealed no potentially antigenic sequence shared (data not shown). In addition, the B cell epitopes confirmed in our study are not preserved following PfTRAP.

In conclusion, this study describes the naturally acquired antibody response against PvTRAP in three endemic municipalities of the Brazilian Amazon. The IgG immune response was associated with exposure and mainly mediated by cytophilic IgG1 antibodies. A significant proportion of IgG3 responders presented a higher time elapsed since the last malaria episode, which could indicate the participation of anti-PvTRAP specific antibodies in protective immune response. Lastly, beyond the validation of four linear B-cell epitopes within PvTRAP full-length sequence, the low response observed against the peptide epitopes could suggest that the functional Domain II of PvTRAP present conformational epitopes. Therefore, more studies need to be addressed to this unexplored target.

## Data Availability

All datasets generated for this study are included in the manuscript/Supplementary Files.

## Ethics Statement

The studies involving human participants were reviewed and approved by Oswaldo Cruz Foundation Ethical Committee and the National Ethical Committee of Brazil. Written informed consent to participate in this study was provided by the participants' legal guardian/next of kin.

## Author Contributions

AM, JL-J, LP-R, and AR-S: conceptualization. AM, JL-J, RR, and JS-A: formal analysis. AM, IS, and RR: investigation. AM, RR, IS, LB-C, and BB: methodology. BB, LP-R, PT, AR-S, CL-C, and RS: sampling and resources. AM, JL-J, and RR: writing—original draft. LP-R, CD-R, CL-C, AR-S, and PT: writing—review and editing.

### Conflict of Interest Statement

The authors declare that the research was conducted in the absence of any commercial or financial relationships that could be construed as a potential conflict of interest.

## Supplementary Material

Figure S1 Reactivity index (RI) of non-exposed individuals (controls), *P. vivax, P. falciparum*, and non-infected exposed individuals. The reactivity index presented no statistical significance among the malaria exposed groups independently of infection status. The reactivity index of non-exposed individuals was significantly lower than exposed individuals (^***^*p* < 0.0001), *P. falciparum* (^***^*p* < 0.0001), and *P. vivax* (^***^*p* < 0.00001).
