# Supplementary figures and images for: Antibody Responses Against Plasmodium vivax TRAP Recombinant and Synthetic Antigens in Naturally Exposed Individuals From the Brazilian Amazon
Source: Front Immunol. 2019 Sep 20;10:2230. doi: 10.3389/fimmu.2019.02230 (PMC6763564; doi:10.3389/fimmu.2019.02230)

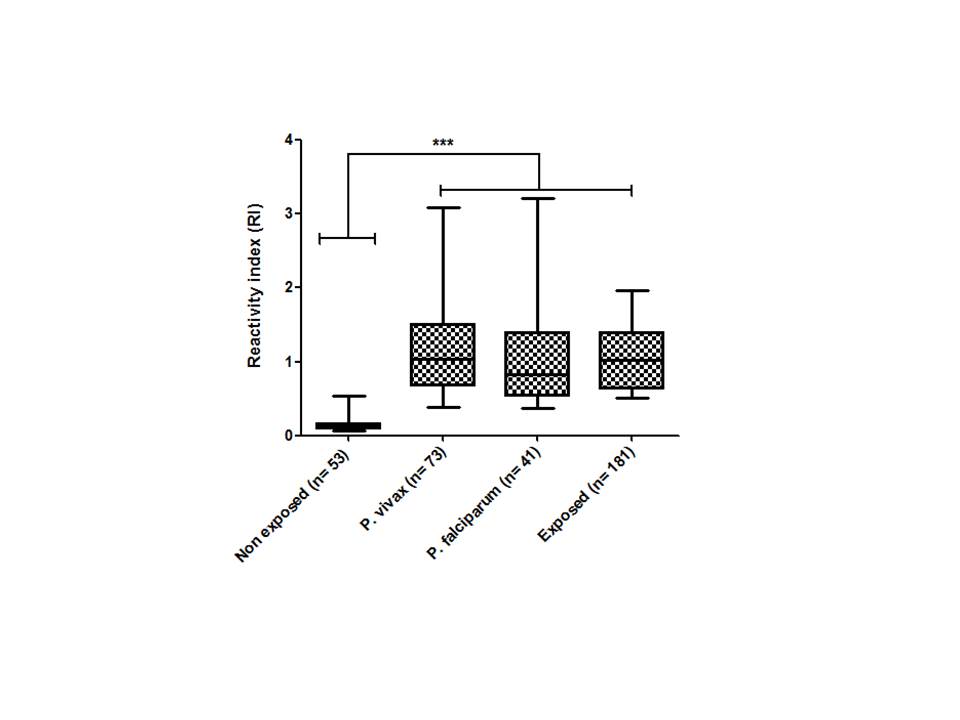

Supplement: Figure S1 — Reactivity index (RI) of non-exposed individuals (controls), P. vivax, P. falciparum, and non-infected exposed individuals. The reactivity index presented no statistical significance among the malaria exposed groups independently of infection status. The reactivity index of non-exposed individuals was significantly lower than exposed individuals (***p < 0.0001), P. falciparum (***p < 0.0001), and P. vivax (***p < 0.00001). [file Image_1.JPEG]
